# Imaging Findings in Pediatric Accessory Cranial Sutures using 3D CT Reconstruction: Fracture or Rudimentary Suture

**DOI:** 10.7759/cureus.42820

**Published:** 2023-08-01

**Authors:** Sirin Falconi, Nagy Laszlo, Roy Jacob

**Affiliations:** 1 Surgery, School of Medicine, Texas Tech University Health Sciences Center, Lubbock, USA; 2 Pediatric Surgery, Texas Tech University Health Sciences Center, Lubbock, USA; 3 Radiology, Texas Tech University Health Sciences Center, Lubbock, USA

**Keywords:** pediatrics, rural neurosurgery, neuroradiology, 3d ct scan, cranial fracture, accessory suture

## Abstract

*Objective:* Although accessory sutures are considered to be relatively rare, the consequences of a missed diagnosis are profound. Distinguishing between accessory sutures and cranial fractures can be difficult, especially in cases of suspected non-accidental trauma. High-resolution imaging is the best way to discern between two- and three-dimensional computerized tomography (3D CT) is considered the preferred method for evaluation. The goal of this study was to determine the impact of 3D CT scans in distinguishing between accessory sutures and cranial fractures in suspected child abuse cases in a rural community and the importance of early detection in such cases, as well as call attention to the consequences of initial misinterpretation.

*Materials and methods:* The researchers conducted a retrospective chart review of all pediatric patients diagnosed with cranial fractures (265 in total) at University Medical Center between May 30, 2016, and May 30, 2021. Initial computed tomography (CT) scans and subsequent 3D CT scans were evaluated for each patient that fit the inclusion criteria, 13 in total. Patients were then categorized into two groups based on the final diagnosis on the radiology report: accessory cranial suture or cranial fracture. Once these patients were identified, the etiology and structural components of each were evaluated, and the key differences were highlighted.

*Results:* Our results showed that, of the 11 cases of suspected non-accidental trauma, six were finally diagnosed with accessory sutures with the use of 3D CT scans, and of those six, four were diagnosed with cranial fractures from the initial CT scan report due to a similar presentation and asymmetric nature.

*Conclusion:* Discerning between fracture and accessory suture is essential in evaluating pediatric patients presenting with signs of cranial fracture due to the increased risk of misinterpretation that can lead to severe legal consequences considering that cranial suture variants may mimic intentional injury and be mistaken for child abuse, causing significant distress for patients and their families.

## Introduction

Cranial sutures are defined as bands of fibrous tissue that hold the cranial bones together and maintain a state of remodeling from infancy through early adulthood to allow the skull to grow and accommodate the developing brain [1,2]. The incidence of accessory sutures, or rudimentary sutures, is considered to be relatively low [1,3]. A study showed that out of 25,000 skulls analyzed post-mortem, only three were found to have the anomaly [3]. While new studies show that the prevalence of these sutures is estimated to be approximately one in every 4000 to 8000 individuals [4]. However, due to the lack of clinical symptoms, the true prevalence in the present population remains unknown [4]. Several studies suggest that accessory sutures are formed from two different ossification centers positioned one above the other, uniting and ossifying radially instead of a single center [3,5]. Incomplete ossification is not rare in these cases, and membranous regions are common after birth, making children the most commonly affected population. Therefore, distinguishing between these unossified regions and fractures can be difficult, especially in the absence of soft tissue swelling [3]. Cases of misdiagnosis are common and carry significant consequences [4,6,7]. Cranial fractures in child abuse are challenging to confirm since normal cranial suture variants mimic intentional injury [4,8,9]. In these cases, a multidisciplinary approach, including clinical presentation and symptomatology, is imperative. Diastasis of sutures could be the only evidence of abusive head trauma; however, it can also be mistaken for a physiologically unfused calvarial state [9,10].

Plain filmography of the skull is routinely obtained as the first method for identifying cranial fractures in adults, but in children, this imaging method is insufficient due to the increased presence of accessories in sutures that can be mistaken for skull fractures [6,11]. CT has replaced plain film in the evaluation of acute pediatric head injuries, and 3D CT imaging is considered the preferred method for the evaluation of cranial fractures and accessory cranial sutures due to increased accuracy in assessing cranial anatomy [4,6,12]. In addition, multidetector CT uses careful size-based kilovolt and milliampere settings to acquire a diagnostically useful dataset. Fine sutures and fractures are difficult to visualize in single planes; therefore, thin-section volumetric acquisition is preferred [13]. Although fractures are difficult to distinguish from sutures, some characteristics can be used to differentiate. Fractures have sharp, non-sclerotic borders and can bifurcate; they can cause diastasis of the sutures, and they include indirect signs such as tissue swelling. On the other hand, sutures tend to join other sutures; they do not cause diastasis of other sutures; their diameter does not change; they are usually symmetric; and they have a “zigzag” or interdigitating pattern with sclerotic borders [8,13]. Follow-up studies with higher-resolution imaging are vital to differentiate a fracture from an accessory suture at the time of diagnosis [11]. When using CT scans, a fracture will show evidence of healing or sclerosis in two or three months [11]. Regardless, repeat imaging is necessary to differentiate between the two and come to the right diagnosis [4,6].

In the past, many studies evaluated the differences between accessory sutures and cranial fractures, but studies evaluating these occurrences in suspected non-accidental trauma in a rural community are limited in sample size and lack evaluation of new imaging techniques, such as 3D CT scanning [11,13,14]. This study aims to determine the impact of 3D CT scanning in distinguishing between accessory suture and cranial fracture in suspected child abuse cases in rural communities and the importance of early detection in such cases, as well as call attention to the consequences of initial misinterpretation.

## Materials and methods

Patient selection

After obtaining institutional review board (IRB) approval for the protection of human subjects (IRB #: L22-083; 03/30/22), we conducted a retrospective chart review of children from ages 0 to 3 years with a diagnosis of cranial fracture or cranial suture over five years at University Medical Center between May 30, 2016, and May 30, 2021. A search of the radiologic database for patients with a diagnosis of cranial fracture or cranial suture was performed, yielding 265 total patients. Only patients who had a diagnosis of cranial fracture or cranial suture and 3D CT imaging were included in this study. Patients were excluded if they had poor-quality scans. Thirteen children were ultimately included in our study who met all the inclusion criteria. 

Covariates 

The chief complaint, mechanism of injury as described by a parent or legal guardian, clinical exam findings, and imaging findings were collected for each patient. Mechanisms of injury were categorized as motor vehicle collision (MVC), fall from height, soft spot on head, suspected abuse, and others, which included dog bite and crush injury. Exam findings focused on the neurological and head, ears, eyes, nose, and throat (HEENT) exams. Data was collected on initial CT imaging and subsequent 3D CT scanning. Following imaging, patients were divided into three separate groups based on initial interpretation: the first group included patients that were diagnosed with cranial fracture by both CT and 3D CT imaging, the second group included patients that were diagnosed with cranial suture by both CT and 3D CT imaging; and finally, the third group included patients that were initially diagnosed with cranial fracture by CT imaging and subsequently had cranial sutures following 3D CT scanning. 

## Results

Patients with accessory suture versus cranial fracture 

A total of 13 patients were included in this study: six were ultimately diagnosed with accessory sutures, and seven were diagnosed with cranial fractures. Eleven out of 13 were initially treated as non-accidental trauma cases. The age at presentation ranged from 2 to 14 months, with a mean of 7.5 months. Table 1 describes each patient according to covariates, age, race, and initial diagnosis.

**Table 1:** Presentation of patients with accessory sutures versus cranial fracture

Patient ID	Gender (M=0; F=1)	Age at diagnosis	Ethnicity (Caucasian=0, Asian/Pacific Islander=1, African American=2, Hispanic/Latino=3, American Indian=4, Other=5	Chief Complaint	Mechanism of Injury (as described by parent/guardian)	Clinical exam findings	Imaging Finding/Interpretation(Fracture= 0; Accessory Suture= 1; Fracture initially then accessory suture= 3)
1	0	2 months	0	abuse	CPS involvement due to suspicion of child abuse	normal exam	3
2	0	4 months	unknown	underlying medical HX of craniosynostosis, plagiocephaly	plagiocephaly consult	left frontal bulging and severe right occipital flattening, right cranial oblique 14.8 cm, left cranial oblique 12.5 cm	1
3	0	8 months	0	fall of height	fall from height	normal exam	3
4	0	13 months	3	possible posterior scalp abscess and CT findings of fracture	abuse	small scab-like lesion on the occipital region at midline, mild erythema, soft, raised, non-tender	3
5	0	4 months	3	fall from height/abuse	fall from height/head trauma	normocephalic, anterior fontanelle firm, flat, and pulsating; no neurological response	3
6	0	14 months	2	congenital microcephaly and an acyanotic congenital heart defect	upper respiratory symptoms (URI) and left-sided facial paralysis	fontanelles flat, significant clear nasal discharge	1
7	0	11 months	0	soft spot on the head	possible fall from height	area of fluctuance over the left parietal region	0
8	0	1 month	0	abuse	abuse/CPS involvement	normal exam	0
9	1	8 months	3	soft spot on the right side of the skull	suspicious nonaccidental trauma	soft right side of the skull	0
10	1	6 months	3	soft spot after being picked up from daycare	unknown	large right parietal cephalohematoma	0
11	1	12 months	3	soft spot on the left side of the head	unknown	left temporal hematoma	0
12	1	4 months	0	abuse	abuse	bulging fontanelles	0
13	0	11 months	3	abuse	abuse	flat posterior head	0

**Figure 1:**
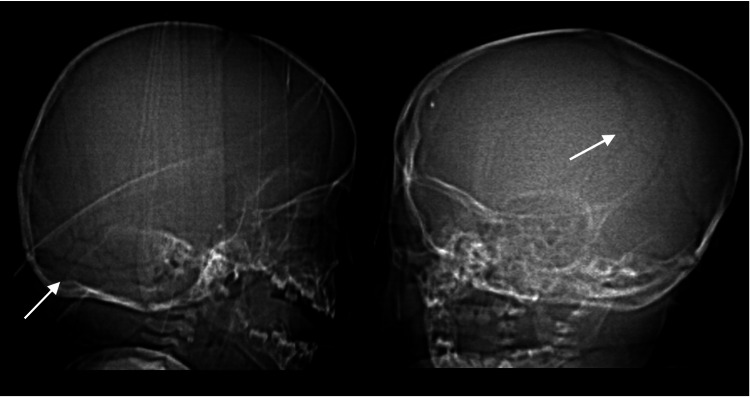
CT scan findings of a patient with accessory cranial suture in a 13-month-old male, sagittal (left) and coronal (right) views. A nondepressed fracture of the right occipital calvarium is present without associated soft tissue swelling.

**Figure 2:**
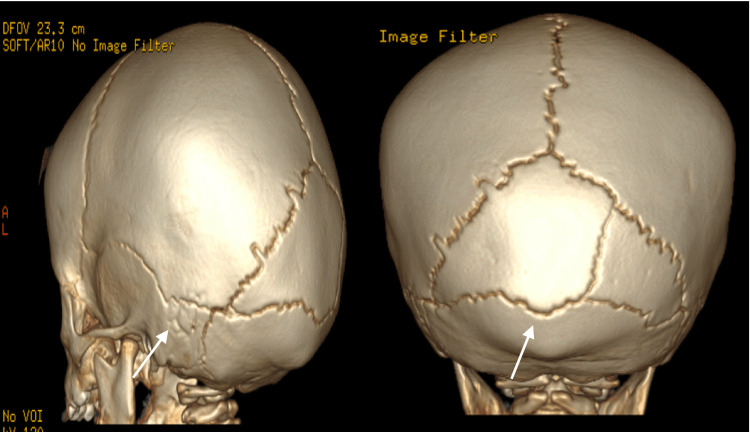
3D CT findings of a patient with accessory cranial suture in a 13-month-old male, sagittal (left) and coronal (right) views. No calvarial fracture was identified; accessory sutures separated the right lateral plate from the medial plate of the interparietal portion of the occipital bone.

Imaging findings comparing CT and 3D CT imaging for the patient with accessory sutures are presented in Table 2 and Figures 1, 2.

**Table 2:** CT imaging vs. 3D CT imaging findings in patients with a final diagnosis of accessory suture

CT Imaging	3D CT Imaging
Non-displaced occipital skull fracture	Extraneous suture from an undetermined cause (Figure *3*)
Possible accessory intraparietal suture in the right parietal bone	Accessory interparietal suture in the right parietal bone, resulting in an asymmetric shape of the calvarium and plagiocephaly
Possible non-depressed skull fracture in the left parietal bone	A linear lucency in the left parietal bone with no overlying soft tissue swelling likely represents an accessory intraparietal suture; a nondisplaced fracture is less likely
Nondepressed fracture of the right occipital calvarium is present without associated soft tissue swelling (Figure *1*)	No calvarial fracture was identified; accessory sutures separated the right lateral plate from the medial plate of the interparietal portion of the occipital bone (Figure *2*)
No depressed calvarial fractures were noted	Incidental note of accessory intraparietal and intra-occipital sutures
Acute subdural hemorrhage overlying bilateral parietal convexities, no mass effect; asymmetric widening of the left atlantooccipital interval, which cannot exclude ligamentous injury; subdural hematomas and cerebral edema; findings worrisome for nonaccidental trauma make further evaluation necessary	There are no calvarial fractures; there is a linear lucency in the left parietal bone, which is felt to represent an accessory intraparietal suture; and there are wormian bones in the occiput. The coronal and sagittal sutures are patent. The lambdoid sutures are patent (Figure *5*)

**Figure 3:**
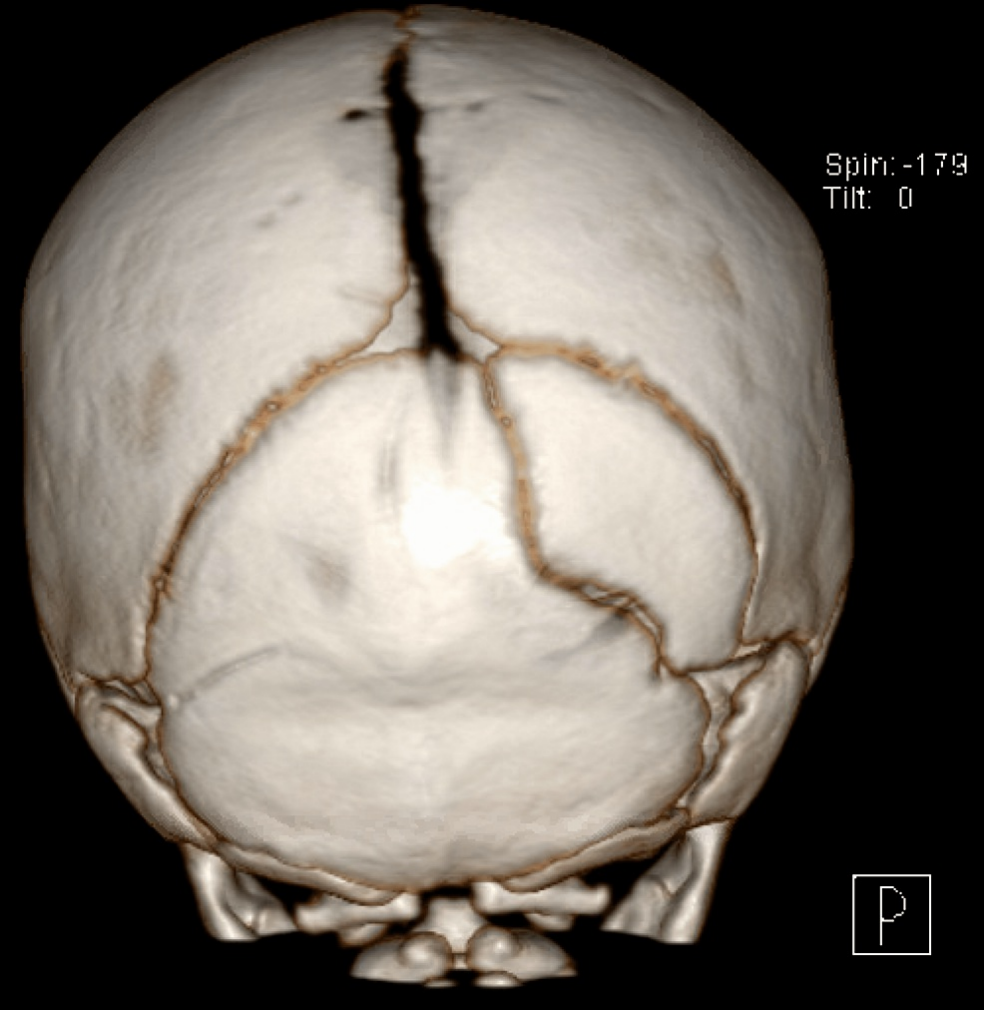
: 3D CT scan findings of accessory cranial sutures in a two-month-old male coronal view. The figure shows an extraneous suture from an undetermined cause.

**Figure 4:**
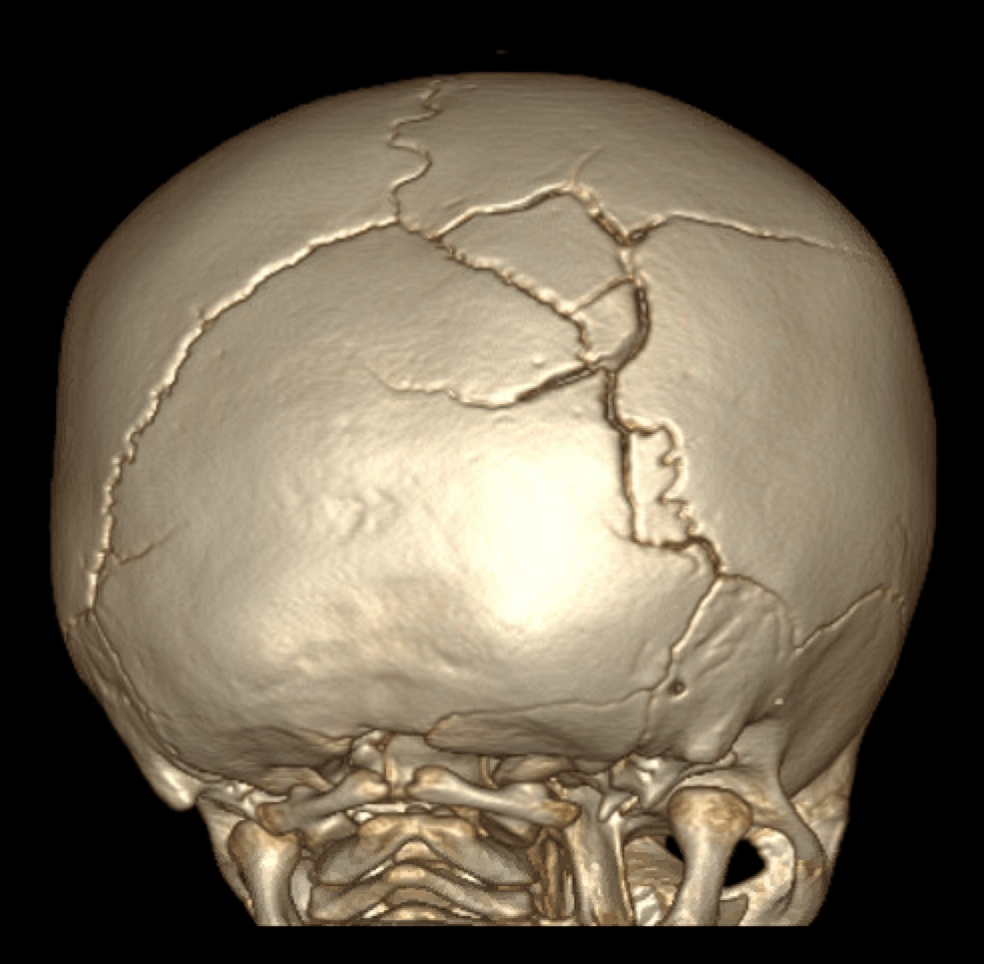
3D CT scan findings of a cranial fracture in a six-month-old female coronal view. The figure shows a fairly nondisplaced fracture of the right parietal and temporal bones, minimally extending into the right occipital bone; it is associated with overlying soft tissue swelling; it crosses suture lines and widens approaching the lambdoid suture line; no significant sclerotic edges are noted, signifying the recent manifestation requiring time for sclerosis.

Figures 3, 4 compare 3D CT scan findings of accessory cranial suture to cranial fracture in two of our patients, highlighting the differences and further demonstrating the importance of high-resolution imaging. An intraoccipital suture can be seen in Figure 3; it merges with the adjacent lambdoid suture and does not change in size; specifically, it does not widen approaching the lambdoid suture; sclerotic edges are apparent along the entire length; it does not cross suture lines. A fairly nondisplaced fracture of the right parietal and temporal bones, minimally extending into the right occipital bone, is visualized in Figure 4; it is associated with overlying soft tissue swelling; it crosses suture lines and widens approaching the lambdoid suture line; no significant sclerotic edges are noted, signifying the recent manifestation requiring time for sclerosis.

**Figure 5:**
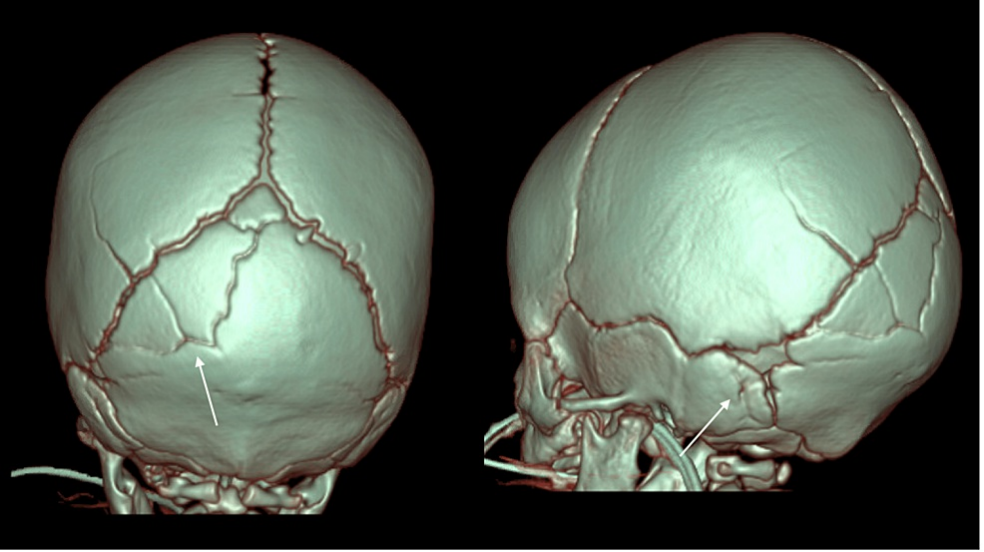
3D CT scan findings of accessory cranial suture with concomitant TBI in a 4-month-old male coronal (left) and sagittal (right) views. There are no calvarial fractures; there is a linear lucency in the left parietal bone, which is felt to represent an accessory intraparietal suture; and there are wormian bones in the occiput. The coronal and sagittal sutures are patent. The lambdoid sutures are patent.

On the other hand, Figure 5 demonstrates the presence of an accessory suture in a patient presenting with a traumatic brain injury (TBI). The 3D CT scan shows no calvarial fractures, linear lucency in the left parietal bone representing an accessory intraparietal suture, and wormian bones in the occiput. 

## Discussion

Accessory sutures are considered rare findings; however, since they are asymptomatic, their true prevalence is not known [4]. To further complicate matters, rudimentary sutures are often mistaken for cranial fractures due to their high resemblance [4,5,7,8,9,11,14,15]. The replacement of plain films by CT scanning decreased the incidence of misdiagnosis between the two; however, additional imaging may be necessary to differentiate questionable cases [11]. Imaging misinterpretation can have severe consequences, especially in suspected non-accidental trauma cases such as child abuse [4,12]. This study aimed to determine the impact of 3D CT scanning in distinguishing between accessory sutures and cranial fractures in suspected child abuse cases in a rural community by evaluating initial CT imaging and comparing it to subsequent 3D CT reconstruction. We also wanted to highlight the importance of early detection in such cases and call attention to the consequences of initial misinterpretation. Our results showed that out of the 13 cases included in our study, 11 were initially treated for suspected non-accidental trauma. Of the six cases with a final diagnosis of accessory suture, four were initially diagnosed and treated for cranial fractures. One of these cases was initially treated at an outside facility before being transferred to our medical center for further evaluation with 3D CT imaging. Furthermore, three out of the four children underwent a skeletal survey to further evaluate for child abuse. 

As determined by Choudhary et al., 3D CT reconstruction has the highest yield of recognition of accessory sutures and can give a definitive diagnosis [16,17]. This is because on CT, it may be difficult to differentiate between suture and fracture on an axial plane alone, and magnetic resonance imaging (MRI) sutures are difficult to visualize, making 3D CT the most accurate imaging method [16]. In our case, 3D CT reconstruction was found to be vital in the final diagnosis of accessory sutures. 3D CT imaging allowed for better visualization of the entire cranium in a relatively short amount of time. No misdiagnosis was made using 3D CT reconstruction. Accessory sutures were recognized immediately by evaluating the specific characteristics that distinguish them from fractures. Rudimentary sutures were found to have a linear lucency with no associated soft tissue swelling or subdural hematoma; no skull depression or bulging fontanelles were found (Table 3). Previous studies showed similar findings distinguishing accessory sutures from cranial fractures with the presence of specific characteristics such as a zigzag pattern with sclerotic borders, no associated diastasis, merging with the adjacent suture, often bilateral and symmetric, and no soft tissue swelling [4,10,14,16,17]. 

**Table 3:** Accessory suture vs. cranial fracture imaging findings

Accessory suture	Cranial fracture
Linear lucency	Depressed fracture
No soft tissue swelling	Associated extracranial soft tissue
No skull depression	Associated scalp contusion
Unremarkable exam findings	Associated subdural hematoma
Fuses with the adjacent suture	Associated overlying soft tissue swelling
Sclerotic borders	Bulging fontanelles
Does not cross suture lines	Crosses suture lines
	Widening of fracture line

As demonstrated by Sanchez et al. and our study, 3D CT imaging can better evaluate cases with inconclusive results in a timely manner [4,16,17]. The availability of 3D CT images is also particularly important when pediatric neuroradiology experience is limited [17]. 3D CT reconstruction has been found to have a sensitivity of 97.7% in the detection of not only normal variants, such as accessory sutures and wormian bones but also diastatic skull fractures [17]. Before the implementation of 3D CT reconstruction, the only way to conclusively discriminate between cranial fracture and rudimentary suture was by follow-up CT imaging to evaluate for evidence of healing or sclerosis two or three months after the first presentation [11]. During that time, these cases were treated as cranial fractures. If non-accidental trauma was suspected, CPS involvement and, thus, severe legal consequences were implemented. In our study, CPS was involved in three out of the six cases of accessory sutures. Complementary studies were initiated, including a skeletal survey, a head-to-toe assessment, an ophthalmology consult, and an initial parent or legal guardian investigation. Following 3D imaging, two cases were dropped, preventing further distress for the patient and family. One of the children found to have accessory sutures was also diagnosed with TBI and, unfortunately, passed away soon after admission due to complications from his injuries (Figure 4). In this case, 3D reconstruction helped further evaluate the cranium while prompting CPS involvement and an investigation of the family. 3D CT imaging not only prevents misdiagnosis but also further consolidates CT scan findings, allowing indisputable proof in difficult cases of child abuse. 

Limitations 

Our study had several limitations. The major limitation is the limited sample size. The methodological constraints and small sample size limit the study’s generalizability. While we reviewed a total of 265 records, not enough data points were found. Additionally, 3D CT reconstruction was not used in many patients; therefore, some patients might have been excluded based on a lack of imaging. Future studies should be broader and include multi-center studies to further increase the amount of data. Finally, one major limitation of our study was its retrospective nature. We were limited by the fact that we had to rely on previously collected data and were not able to confirm it. 

## Conclusions

3D CT scanning may help decrease the occurrence of misdiagnosis and further improve the detection of rudimentary sutures in rural communities, especially in equivocal cases. Discerning between fracture and accessory suture is essential in evaluating pediatric patients presenting with signs of cranial fracture due to the increased risk of misinterpretation that can lead to severe legal consequences considering that cranial suture variants may mimic intentional injury, and be mistaken for child abuse, causing significant distress for patients and their families.
